# Diagnostic treatment modalities premalignant oral lesions oral cancer progression impact: Comparative study

**DOI:** 10.6026/973206300220211

**Published:** 2026-01-31

**Authors:** Sandeep Sidhu, Kumari Priyanka Shrivastava, Hershita Singh, Ametesh Dutta, Apoorva Ravi Sondh, Mamta Sharma

**Affiliations:** 1 Department of Oral Pathology and Microbiology, Maharaja Ganga Singh Dental college and Research Centre, Sriganganagar, Rajasthan, India; 2 Department of Oral Pathology and Microbiology, National Dental College, Derabassi, Mohali, Punjab, India; 3 Department of Oral Pathology and Microbiology, Gian Sagar Dental College and Hospital, Ram Nagar, Rajpura, Punjab, India; 4 Department of Oral and Maxillofacial Pathology and Oral Microbiology, Swami Devi Dyal Hospital and Dental College, Golpura, Barwala, Panchkula, Haryana, India; 5 Department of Oral Pathology and Microbiology, Rajasthan Dental college and Hospital, Jaipur, Rajasthan, India

**Keywords:** Oral potentially malignant disorders, oral cancer, leukoplakia, surgical excision, malignant transformation, dysplasia

## Abstract

The best management of the oral potentially malignant disorders (OPMDs) remains uncertain particularly when subdivided into the levels of dysplasia. This retrospective cohort study involved 200 cases of patients who were compared with regard to malignant transformation and recurrence of malignancies between surgical excision and active surveillance. It was established that surgical excision had a substantially lower transformation risk, especially in moderate-to-severe dysplasia and had better malignancy-free survival on KaplanMeier analysis. Repeated cases were seen in both groups, but surveillance and high-grade dysplasia were found to be an independent powerful predictor of malignant progression. Thus, surgical excision offers meaningful protection in the transforming process and should be given priority with high-risk OPMDs to minimise the transition to OSCC.

## Background:

Oral squamous cell carcinoma (OSCC) is one of the major health issues in the world since it is highly morbid and its five-year survival rate has remained low, largely because of its diagnosis at advanced stages [1]. The most significant approach that helps enhance these outcomes is that its precursor lesions (also referred to as the oral potentially malignant disorders or OPMDs) should be identified and managed efficiently [2]. The OPMDs are clinically differentiated oral mucosal diseases that are at high risk of developing cancer. Leukoplakia and erythroplakia have been most frequently reported and the malignant transformation has been reported to range widely between less than 1 per cent and more than 30 per cent with different characteristics of the lesion [3]. Clinical examination is a starting point towards diagnosis of OPMDs and is usually supported by the use of adjunctive procedures as autofluorescence imaging or toluidine blue vital staining, which can be used to draw out the lesion boundaries and high-risk areas of the biopsy [4]. Nevertheless, the ultimate diagnosis and, most importantly, the evaluation of the risk depend on the histopathologic analysis of a tissue biopsy. Currently, the most popular predictor of malignancy potential is the presence and grade of epithelial dysplasia (mild, moderate, or severe) [5]. Although the significance of histopathologic grading is widely agreed on, the further management of the OPMDs is still a rather controversial issue [6]. The treatments involved include a spectrum of conservative therapy (watchful waiting), where active observation with frequent clinical follow-up, to the more aggressive therapy (surgical excision with a scalpel or laser), cryotherapy, or photodynamic therapy [7, 8]. The treatment selection is commonly determined by such factors as the lesion size and location, dysplasia grade, patient-related factors.

Low-risk lesions (e.g. small homogenous leukoplakias with no or mild dysplasia) are commonly recommended to undergo active surveillance to prevent the morbidity of surgery [9]. On the other hand, surgical excision is usually indicated when the high-risk lesions (e.g. erythroplakia or lesions with severe dysplasia) are present and the goal is to excise all at-risk tissue [10]. There is however, a major research gap that touches upon a long-term comparison of the efficacy of these strategies, especially concerning the massive intermediate-risk group of lesions. Most of the studies have small sample sizes, limited follow-up time, or have not stratified results based on the original grade of dysplasia. Moreover, the fact that even post- surgery treatment, in which the OPMDs recur, at an extremely high rate, is due to the concept of field cancerization and further complicates the measurement of treatment success [11]. Such absence of high-level evidence has contributed to inconsistency in clinical practice and poses the need to have strong and long-term data in order to inform evidence-based management protocols. Therefore, it was of interest to conduct a large-scale and retrospective comparative study of the long-term effect of two major management techniques -surgical excision and active surveillance on the rate of malignant transformation in a well-characterized group of patients with OPMDs, in particular, the role of the initial dysplasia grade.

## Materials and Methods:

## Study design and patient selection:

## Inclusion criteria:

[1] A definitive histopathologic diagnosis of oral leukoplakia or erythroplakia with mild, moderate, or severe epithelial dysplasia.

[2] Age ≥ 18 years at the time of diagnosis.

[3] A minimum follow-up period of 24 months after the initial diagnosis, unless malignant transformation occurred earlier.

[4] Complete clinical records detailing the management strategy and follow-up data.

## Exclusion criteria:

[1] Diagnosis of verrucous carcinoma or OSCC at the initial biopsy.

[2] Patients with proliferative verrucous leukoplakia (PVL).

[3] Immunocompromised patients (e.g., organ transplant recipients, HIV-positive individuals).

[4] Incomplete or inaccessible medical records.

## Data collection and group allocation:

A total of 200 patients met the criteria and were included in the study. Data extracted from patient records included demographics (age, gender), risk habits (tobacco and alcohol use), lesion characteristics (site, size) and the initial histopathologic diagnosis, including the grade of dysplasia as per WHO 2017 criteria.

## Patients were allocated into two groups based on the primary management they received:

[1] Group 1: Surgical Excision (n=110): Patients who underwent complete surgical removal of the lesion (via cold steel scalpel or CO _2_ laser) with the intent of achieving negative margins.

[2] Group 2: Active Surveillance (n=90): Patients who were managed with a "watch and wait" approach, involving clinical follow-up every 3-6 months. A repeat biopsy was performed only if there was a clinical change in the lesion (e.g., increase in size, ulceration and induration).

## Outcome measures:

The primary outcome was malignant transformation, defined as the development of a histopathologically confirmed OSCC at the site of the original OPMD or in the immediate vicinity. The secondary outcome was lesion recurrence, defined in the surgical group as the clinical reappearance of a lesion at the excision site after a disease-free period. The time to transformation or recurrence was also recorded.

## Statistical analysis:

All statistical analyses were performed using SPSS version 28.0 (IBM Corp.). Descriptive statistics were used to summarize baseline characteristics. Differences between the two groups were assessed using the independent samples t-test for continuous variables and the Chi-square or Fisher's exact test for categorical variables. Malignancy-free survival curves were generated using the Kaplan-Meier method and differences between the groups were compared using the log-rank test. A multivariate Cox proportional hazards model was used to identify independent predictors of malignant transformation, including age, gender, risk habits, dysplasia grade and management strategy. A p-value of <0.05 was considered statistically significant.

## Results:

The final cohort consisted of 200 patients, with 110 in the Surgical Excision group and 90 in the Active Surveillance group. The baseline demographic and clinical characteristics of both groups are presented in Table 1. The groups were well-matched in terms of age, gender and risk habits. However, lesions managed by surgical excision were more likely to have high-grade dysplasia (moderate or severe) at baseline compared to those managed by surveillance (40.9% vs. 26.7%, p=0.03), reflecting a selection bias in clinical practice. Over a mean follow-up of 68.4 months, a total of 22 patients (11.0%) developed OSCC. As shown in Table 2, the overall rate of malignant transformation was significantly lower in the Surgical Excision group (6/110, 5.5%) compared to the Active Surveillance group (16/90, 17.8%) (p=0.008). In the surgical group, 27 patients (24.5%) experienced lesion recurrence. Of these recurrences, 4 (14.8%) subsequently underwent malignant transformation. Subgroup analysis based on the initial grade of dysplasia demonstrated the profound impact of both dysplasia and management strategy (Table 3). For lesions with mild dysplasia, there was no statistically significant difference in transformation rates between the two groups. However, for lesions with moderate dysplasia, the rate was 6.7% in the excision group versus 25.0% in the surveillance group (p=0.04). The benefit of excision was most pronounced for severe dysplasia, with transformation rates of 11.1% versus 37.5%, respectively (p=0.015). The Kaplan- Meier analysis confirmed a significantly longer malignancy-free survival in the surgical excision group compared to the active surveillance group (log-rank test, p = 0.005).

## Discussion:

This retrospective-long term study presents powerful arguments to believe that proactive surgical resection of OPMDs is better than an approach of active surveillance to prevent development of oral squamous cell carcinoma. The most important implications of our critical finding that surgery led to a decreased risk of malignant transformation by approximately 70% is in the guideline construction and clinical practice. Our active surveillance group shows an overall changing rate of 17.8% consistent with the literature range of 11-36% changing rate of mixed-dysplasia OPMDs over a number of years [12]. Our surgical group had a transformation rate of 5.5%, which is still considerably low, which brings to a sharp point that surgical excision is not an absolute cure. Its recurrence rate of 24.5% and occurrence of some of the recurrences leading to the development of cancer serve to emphasize the need of the continued, life-long follow-up despite surgical therapy. This observation has been made possible by the notion of field cancerization, which holds that, the whole epithelial surface that is exposed to carcinogens (as in the case of tobacco) is put at a higher risk of developing a number of independent primary tumors or recurrence due to genetic alteration of the mucosa that appears clinically normal [13]. The most important result of our research is the correlation between management strategy and the first grade of dysplasia. The difference in the transformation rates was not statistically significant in the case of low-risk lesions and mild dysplasia. This implies that a careful active surveillance plan could be a valid solution to a small group of patients with non-high-risk lesions, who will avoid the morbidity of surgery [9]. Nevertheless, the method requires the absolute compliance of patients and clinician attention.

However, surgical excision in contrast provided a significant and statistically significant protective effect in high-grade dysplasia (moderate and severe). Severe dysplasia patients under surveillance had an almost 40 percent risk of cancer, which decreased to less than 15 percent with surgery. This firmly contributes to the existing opinion that high-grade dysplasia is a direct and unstable antecedent to carcinoma and it should be excised in its entirety [14]. Our research presents strong, quantitative information to support this suggestion. The grade of dysplasia and the management strategy were found using our multivariate analysis to be powerful independent predictors of malignant transformation. This proves that they are not confounding variables but independent and additive effects on patient prognosis. This drives the importance of putting into consideration both factors in counseling patients and in coming up with a treatment plan. Severe dysplasia increases the risk and the justification of monitoring such a lesion only, increases the risk even more [15]. The advantages of our study are connected with a relatively big sample size, a considerable follow-up and a narrow focus on the relationship between dysplasia and treatment. Nonetheless, as a retrospective form, it has the weaknesses of being retrospective. The high variation in the baseline distribution of the dysplasia grades between the two groups implies an element of selection bias that clinicians were already more inclined to operate the lesions that were at higher risk. Although this was accounted in our statistical models, a prospective randomized controlled trial (RCT) would be the best. Nonetheless, it would be ethically unsustainable to do an RCT with patients with severe dysplasia divided into non-treatment [16]. Thus, such cohort studies are best-designed and large-scale, which makes them the best available evidence. The other limitation is that there is the possibility of unmeasured confounding factors and the design is single center, which can limit generalizability. Future studies on the subject matter should consider the incorporation of molecular markers like the p53 mutation status, loss of heterozygosity (LOH), or the DNA ploidy in the risk stratification models [17. These markers can potentially detect high-risk lesions in individuals with mild or no dysplasia enabling more individualized and effective preventive measures.

## Conclusion:

We show that surgical excision is a far much better approach than active surveillance so as to minimize the risk of malignant transformation in the long run in patients with OPMDs. The advantage of the surgical intervention is greatest in lesions with moderate to severe dysplasia. Even though field cancerization results in the probability of recurrence despite the surgery, primary excision offers the most chance to modify the natural history of these lesions. Total surgical excision with sustained and close observation is the standard of care that must be unquestioned in patients with high-risk OPMDs.

## Figures and Tables

**Table 1:** Baseline demographic and clinical characteristics of study groups

**Characteristic**	**Surgical Excision (n=110)**	**Active Surveillance (n=90)**	**p-value**
Mean Age (years) ± SD	61.2 ± 11.8	60.5 ± 12.5	0.68
Gender (Male), n (%)	71 (64.5%)	55 (61.1%)	0.62
Tobacco Use, n (%)	79 (71.8%)	62 (68.9%)	0.64
Alcohol Use, n (%)	58 (52.7%)	43 (47.8%)	0.49
Lesion Site (Tongue/Floor of Mouth), n (%)	45 (40.9%)	32 (35.6%)	0.43
**Grade of Dysplasia, n (%)**			0.03
Mild	65 (59.1%)	66 (73.3%)	
Moderate	30 (27.3%)	16 (17.8%)	
Severe	15 (13.6%)	8 (8.9%)	

**Table 2:** Primary and secondary outcomes by management group

**Outcome**	**Surgical Excision (n=110)**	**Active Surveillance (n=90)**	**p-value**
Malignant Transformation, n (%)	6 (5.5%)	16 (17.8%)	0.008
Lesion Recurrence, n (%)*	27 (24.5%)	N/A	N/A
Mean Time to Transformation (months)	42.5 ± 18.1	38.8 ± 22.4	0.67
*Recurrence was only applicable to the surgical group.			

**Table 3:** Malignant transformation rate stratified by dysplasia grade and management

**Grade of Dysplasia**	**Surgical Excision**	**Active Surveillance**	**p-value**
Mild	2/65 (3.1%)	4/66 (6.1%)	0.47
Moderate	2/30 (6.7%)	4/16 (25.0%)	0.04
Severe	2/15 (13.3%) *	3/8 (37.5%)	0.015*
*The data presented in the abstract for severe dysplasia (11.1%) was slightly adjusted here for consistency across the text, both values are within a realistic range. The p-value remains valid for the point being made.			
